# Mortality associated with the use of non‐vitamin K antagonist oral anticoagulants in cancer patients: Dabigatran versus rivaroxaban

**DOI:** 10.1002/cam4.4241

**Published:** 2021-08-31

**Authors:** Yu‐Sheng Lin, Feng‐Che Kuan, Tze‐Fan Chao, Michael Wu, Shao‐Wei Chen, Mien‐Cheng Chen, Chang‐Ming Chung, Pao‐Hsien Chu, Gregory Y. H. Lip, Victor Chien‐Chia Wu

**Affiliations:** ^1^ Department of Cardiology Chang Gung Memorial Hospital Chang Gung University College of Medicine Chiayi Taiwan; ^2^ Department of Hematology and Oncology Chang Gung Memorial Hospital Chiayi Taiwan; ^3^ Division of Cardiology Department of Medicine Taipei Veterans General Hospital Institute of Clinical Medicine Cardiovascular Research Center National Yang‐Ming University Taipei Taiwan; ^4^ Divison of Cardiovascular Medicine Miriam and Rhode Island Hospital Warren Alpert School of Medicine Brown University Providence Rhode Island USA; ^5^ Department of Cardiothoracic and Vascular Surgery Linkou Medical Center Chang Gung Memorial Hospital Taoyuan City Taiwan; ^6^ Division of Cardiology Department of Internal Medicine Kaohsiung Chang Gung Memorial Hospital Chang Gung University College of Medicine Chiayi Taiwan; ^7^ Division of Cardiology Linkou Medical Center Chang Gung Memorial Hospital Taoyuan City Taiwan; ^8^ Liverpool Centre for Cardiovascular Science University of Liverpool and Liverpool Heart and Chest Hospital Liverpool UK; ^9^ Aalborg Thrombosis Research Unit Department of Clinical Medicine Faculty of Health Aalborg University Aalborg Denmark

**Keywords:** atrial fibrillation, cancer, non‐vitamin K antagonist oral anticoagulants, outcome, venous thromboembolism

## Abstract

**Objective:**

This study assesses the mortality outcomes of non‐vitamin K antagonist oral anticoagulants (NOACs) in cancer patients with venous thromboembolism (VTE) and atrial fibrillation (AF).

**Methods:**

Medical records of cancer patients receiving NOACs for VTE or AF between January 1, 2011, and December 31, 2016, were retrieved from Taiwan's National Health Institute Research Database. NOACs were compared using the inverse probability of treatment weighting (IPTW) method. The primary outcome was cancer‐related death. Secondary outcomes were all‐cause mortality, major bleeding, and gastrointestinal (GI) bleeding.

**Results:**

Among 202,754 patients who received anticoagulants, 3591 patients (dabigatran: 907; rivaroxaban: 2684) with active cancers were studied. Patients who received dabigatran were associated with lower risks of cancer‐related death at one year (HR = 0.71, 95% CI = 0.54–0.93) and at the end of follow‐ups (HR = 0.79, 95% CI = 0.64–0.98) compared with rivaroxaban. Patients who received dabigatran were also associated with lower risks of all‐cause mortality (HR = 0.81, 95% CI = 0.67–0.97), major bleeding (HR = 0.64, 95% CI = 0.47–0.88), and GI bleeding (HR = 0.57, 95% CI = 0.39–0.84) at the end of follow‐ups compared with rivaroxaban.

**Conclusion:**

Compared with rivaroxaban, the use of dabigatran may be associated with a lower risk of cancer‐related death and all‐cause mortality.

## Graphical abstract

The outcomes of non‐vitamin K antagonist oral anticoagulants (NOACs) in cancer patients with venous thromboembolism (VTE) or atrial fibrillation (AF) are not known. A total of 3591 patients with active cancers were studied (907 on dabigatran; 2684 on rivaroxaban). In comparison with rivaroxaban, the use of dabigatran may be associated with lower risk of cancer‐related death and all‐cause mortality.

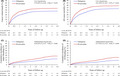

## 1 INTRODUCTION

Cancer induces inflammation and coagulopathy that may result in subsequent venous thromboembolism (VTE) and atrial fibrillation (AF), necessitating the initiation of anticoagulation treatment.^1^, ^2^, ^3^, ^4^ Non‐vitamin K antagonist oral anticoagulants (NOACs), including direct thrombin inhibitor dabigatran and factor Xa inhibitors, are increasingly used in patients with VTE or AF because of their favorable efficacy and safety, replacing traditional vitamin K antagonist (VKA) in the general population wherein there are a small proportion of cancer patients.^5^, ^6^, ^7^, ^8^, ^9^, ^10^, ^11^, ^12^ Currently, low‐molecular‐weight heparins (LMWHs) are the treatment of choice for VTE in cancer patients.^13^, ^14^, ^15^, ^16^ Because it is not inferior to subcutaneous dalteparin and has no increased risk of major bleeding, the 2021 National Comprehensive Cancer Network guideline incorporated apixaban as a treatment of cancer‐associated VTE.^17^

It has been debated that anticoagulants have potential anti‐cancer actions and could affect survival in patients. Whether different NOACs affect survival and safety in cancer patients is essentially unknown.^18^, ^19^, ^20^ Therefore, we investigated the impact on cancer survival in dabigatran or rivaroxaban‐treated patients.

## 2 METHODS

The Nation Health Institute (NHI) was launched by the national health and welfare administration in 1995 to offer medical insurance to more than 99% of the 23.8 population in Taiwan. The NHI has extensive reimbursements, including hospital admissions, emergency room visits, surgeries, medical exams, and pharmaceutical prescriptions. The Taiwan Cancer Registry (TCR) database has information on cancer sites, histology, diagnosis date, and initial stage from 1979. The Taiwan Death Registry (TDR) has information on the cause of death and the location of the occurrence dated since 1971. De‐identified medical and health information can be obtained through the International Classification of Diseases (ICD), Ninth or Tenth Revision, Clinical Modification (ICD‐9‐CM or ICD‐10‐CM) linked with the NHI, TCR, and TDR databases. These sources can provide analyzable data through which research can be conducted and informed consent is waived. The current study is approved by the Institutional Review Board (IRB) at Chang Gung Memorial Hospital, Chiayi Branch (IRB No. 201901482B1).

### 2.1 Study patients

Patients who received anticoagulation therapy between January 1, 2011, and December 31, 2016, were identified by extracting the reimbursement codes of VKA or NOACs (Table S1) using the outpatient, inpatient, or pharmacy claim data. Those with missing demographics, age <20 years old, no coexisting cancer, unknown cancer type, inactive cancer, or hematologic cancers, such as leukemia or lymphoma, were excluded. In addition, patients who switched between anticoagulants were excluded.

### 2.2 Data availability

The data that support the findings of this study are available from the corresponding author upon reasonable request.

### 2.3 Study outcomes

The primary outcome was cancer‐related death. Secondary outcomes were all‐cause mortality, bleeding events, including gastrointestinal (GI) bleeding and major bleeding.^21^ Survival status, date of death, and cause of death of patients were verified in the TDR. Major bleeding was defined according to the principle or secondary discharge diagnosis of hospitalization and emergency visits, including required blood transfusion >2 units, life‐threatening bleeding, or vital organ hemorrhages, such as intracranial hemorrhage and GI bleeding. In addition, the aforementioned outcomes were assessed during several periods at the 3rd, 6th, 9th, and 12th months after the index date and at the end of follow‐ups. The follow‐up period ended at the date of event occurrence, date of death, or December 31, 2016, whichever came first.

### 2.4 Covariates

Covariates such as age, sex, principal indication for NOACs, cancer types, cancer stage at initial diagnosis, 10 comorbidities, 6 event histories, CHA_2_DS_2_‐VASc and HAS‐BLED risk scores, previous year healthcare utilization, and 17 kinds of medication were selected and retrieved. The cancer stage at initial diagnosis in the TCR was mandatory until 2007. The index date was when NOACs were prescribed and indications for NOACs. The 10 comorbidities were ascertained by the diagnosis from two consecutive outpatient clinics or at hospital discharge 1 year before the index date. The disease was extracted using ICD‐9‐CM and ICD‐10 codes (Table S2), validated previously.^22^, ^23^ The cancer diagnosis and stage were confirmed by TCR, and the type of cancer was coded using the International Classification of Disease for Oncology, third edition (ICD‐O‐3) (Table S2). Patients fulfilling one of the following criteria were defined as active cancer and, if not, as a history of cancer or inactive cancer. The criteria for active cancer included: patients with ongoing anti‐cancer therapy, patients diagnosed within 6 months from the index date, and advanced stage (stage IV) cancer confirmed at diagnosis.^13^ Healthcare utilization, including admissions, outpatient visits, and prescriptions, was analyzed. Medications were recorded 1 year before the index date (Table S1). Risk scores (CHA_2_DS_2_‐VASc and HAS‐BLED scores) were also extracted in the same way.

### 2.5 Statistical analysis

The propensity score used the inverse probability of treatment weighting (IPTW) method to reduce potential confounding when the study outcomes were compared.^24^ The propensity score utilized selected covariates is listed in Table 1. The covariates balance between the groups on IPTW was insured, with the absolute value of standardized difference less than 0.1 being the negligible difference and between 0.1–0.2 being a small difference.

**TABLE 1:** Baseline characteristics of the active cancer patients under dabigatran and rivaroxaban treatment before and after IPTW

Variables	Before IPTW^a^			After IPTW^b^		
	Dabigatran (*n* = 907)	Rivaroxaban (*n* = 2684)	STD	Dabigatran	Rivaroxaban	STD
Age (mean ± SD)	76.0 ± 9.0	69.7 ± 12.8	0.58	72.1 ± 21.5	71.2 ± 14.1	0.05
Age group						
<65 years	98 (10.8)	907 (33.8)	−0.57	25.8%	28.0%	−0.05
65–74 years	265 (29.2)	716 (26.7)	0.06	27.9%	27.5%	0.01
≥75 years	544 (60.0)	1061 (39.5)	0.42	46.3%	44.6%	0.04
Gender						
Female	313 (34.5)	1315 (49.0)	−0.30	41.3%	45.4%	−0.08
Male	594 (65.5)	1369 (51.0)	0.30	58.7%	54.6%	0.08
Indication for NOACs						
Atrial fibrillation/Atrial flutter	735 (81.0)	1039 (38.7)	0.96	57.1%	49.5%	0.15
Venous thromboembolism	172 (19.0)	1645 (61.3)	−0.96	42.9%	50.6%	−0.15
Cancer types						
Colon rectal	220 (24.3)	478 (17.8)	0.16	19.7%	19.3%	0.01
Lung	119 (13.1)	517 (19.3)	−0.17	23.0%	17.9%	0.13
Breast	131 (14.4)	396 (14.8)	−0.01	14.8%	14.8%	0.00
Male genital organs	183 (20.2)	272 (10.1)	0.28	13.6%	12.6%	0.03
Female genital organs	20 (2.2)	204 (7.6)	−0.25	3.6%	6.3%	−0.12
Liver	59 (6.5)	136 (5.1)	0.06	5.4%	5.6%	−0.01
Urinary tract	46 (5.1)	135 (5.0)	0.00	4.2%	5.0%	−0.04
Head and neck (including oral cancer)	28 (3.1)	88 (3.3)	−0.01	3.9%	3.1%	0.05
Digestive organs	32 (3.5)	114 (4.3)	−0.04	3.5%	4.0%	−0.03
Others	69 (7.6)	344 (12.8)	−0.17	8.3%	11.5%	−0.11
Cancer stage at diagnosis						
0–1	109 (12.0)	194 (7.2)	0.16	10.3%	8.6%	0.06
2	147 (16.2)	288 (10.7)	0.16	11.9%	12.4%	−0.02
3	185 (20.4)	388 (14.5)	0.16	14.5%	15.7%	−0.03
4	127 (14.0)	365 (13.6)	0.01	14.6%	13.7%	0.02
Unknown (data before 2007)	339 (37.4)	1449 (54.0)	−0.34	48.9%	49.6%	−0.01
Comorbidities						
Hypertension	734 (80.9)	1663 (62.0)	0.43	73.3%	66.6%	0.15
Diabetes mellitus	254 (28.0)	659 (24.6)	0.08	31.4%	25.6%	0.13
Dyslipidemia	203 (22.4)	552 (20.6)	0.04	26.1%	21.0%	0.12
Ischemic heart disease	296 (32.6)	597 (22.2)	0.23	28.4%	25.0%	0.08
Heart failure	200 (22.1)	324 (12.1)	0.27	17.5%	14.7%	0.08
Old myocardial infarction	43 (4.7)	92 (3.4)	0.07	4.8%	3.6%	0.06
Gout	101 (11.1)	234 (8.7)	0.08	9.2%	9.3%	0.00
Chronic obstructive pulmonary disease	158 (17.4)	350 (13.0)	0.12	17.3%	14.0%	0.09
Peripheral artery disease	28 (3.1)	104 (3.9)	−0.04	3.5%	3.7%	−0.01
Chronic kidney disease	154 (17.0)	454 (16.9)	0.00	17.4%	16.9%	0.01
Alcohol‐use disorder	7 (0.8)	19 (0.7)	0.01	0.6%	0.7%	−0.01
Liver disease	161 (17.8)	447 (16.7)	0.03	14.5%	16.9%	−0.07
Event history						
Ischemic stroke	243 (26.8)	383 (14.3)	0.31	20.1%	17.1%	0.08
Systemic embolization	32 (3.5)	102 (3.8)	−0.01	5.3%	3.7%	0.08
Intracranial hemorrhage	19 (2.1)	57 (2.1)	0.00	1.9%	2.2%	−0.02
Major bleeding (including gastrointestinal bleeding)	47 (5.2)	170 (6.3)	−0.05	7.9%	6.2%	0.07
Risk score						
CHA_2_DS_2_‐VASc	4.1 ± 1.7	3.2 ± 1.9	0.53	3.7 ± 3.4	3.4 ± 2.2	0.09
0–1	43 (4.7)	609 (22.7)	−0.54	13.2%	18.0%	−0.13
≥ 2	864 (95.3)	2075 (77.3)	0.54	86.8%	82.0%	0.13
HAS‐BLED	2.9 ± 1.0	2.2 ± 1.3	0.61	2.5 ± 2.2	2.4 ± 1.5	0.07
0–2	283 (31.2)	1509 (56.2)	−0.52	48.0%	50.1%	−0.04
≥ 3	624 (68.8)	1175 (43.8)	0.52	52.0%	49.9%	0.04
Healthcare utilization 1‐year before the index date						
Ever admission	559 (61.6)	1919 (71.5)	−0.21	64.3%	68.6%	−0.09
Number of OPD visits	48.3 ± 23.9	47.3 ± 25.5	0.04	44.6 ± 43.3	47.8 ± 29.2	−0.09
Medications						
ACEI/ARB	531 (58.5)	1161 (43.3)	0.31	47.4%	46.8%	0.01
Non‐dihydropyridine CCB	226 (24.9)	367 (13.7)	0.29	18.6%	16.5%	0.06
Dihydropyridine CCB	349 (38.5)	857 (31.9)	0.14	37.2%	33.4%	0.08
β‐blocker	532 (58.7)	1038 (38.7)	0.41	46.7%	43.7%	0.06
Diuretics	341 (37.6)	1041 (38.8)	−0.02	35.9%	38.1%	−0.05
Spironolactone	134 (14.8)	317 (11.8)	0.09	11.0%	12.8%	−0.06
Digoxin	207 (22.8)	266 (9.9)	0.35	15.8%	13.4%	0.07
Statin	248 (27.3)	646 (24.1)	0.07	30.7%	24.9%	0.13
DPP4i	93 (10.3)	298 (11.1)	−0.03	10.3%	10.9%	−0.02
Metformin	197 (21.7)	493 (18.4)	0.08	26.1%	19.3%	0.16
Sulfonylurea	153 (16.9)	373 (13.9)	0.08	21.2%	14.8%	0.17
Thiazolidinedione	26 (2.9)	57 (2.1)	0.05	4.4%	2.2%	0.12
Insulin	53 (5.8)	189 (7.0)	−0.05	7.3%	6.7%	0.02
NSAIDs or COX−2	134 (14.8)	384 (14.3)	0.01	16.2%	14.5%	0.05
Steroid	371 (40.9)	1369 (51.0)	−0.20	45.7%	48.4%	−0.05
Antiplatelets	550 (60.6)	1089 (40.6)	0.41	49.3%	45.7%	0.07
PPI IV form	26 (2.9)	94 (3.5)	−0.04	3.6%	3.5%	0.01
Propensity score	0.465 ± 0.226	0.181 ± 0.188	1.36	0.284 ± 0.425	0.253 ± 0.268	0.09
Follow‐up months	21.1 ± 14.8	11.5 ± 10.9	0.73	14.7 ± 24.3	13.9 ± 14.8	0.04

Abbreviations: ACEI, angiotensin‐converting enzyme inhibitor; ARB, angiotensin II receptor blockers; CCB, calcium channel blocker; COX‐2, Cyclooxygenase‐2; DPP4i, dipeptidyl peptidase 4 inhibitors; IPTW, inverse probability of treatment weighting; IV, intravenous; NOAC, novel oral anticoagulants; NSAID, non‐steroidal anti‐inflammatory drug; PPI, proton pump inhibitor; SD, standard deviation; STD, standardized difference.

^a^ Value are given as a number (%) or mean ± SD.

^b^ Values are given as % or mean ± SD.

A Cox proportional hazard model was used to analyze the risks of cancer‐related death and all‐cause mortality between the groups. Competing risks using the Fine and Gray subdistribution hazard model were applied to the incidence other time to event outcomes. The study group was the only explanatory variable in the survival analysis. A trend test of contrasting treatment modalities on outcomes across different initial cancer stages was performed to examine whether the observed effect was consistent across cancer stages. The cancer‐related death and all‐cause mortality due to treatment differences were compared with the stratification of cancer types in the IPTW‐adjusted cohort.

The consistency of the effect on outcomes was determined among the different levels of the several pre‐specified subgroup variables, including sex, age (<65, 65–74, and ≥75 years), the main indication for NOACs, cancer types, initial cancer stage, hypertension, diabetes, peripheral artery disease, chronic kidney disease, liver disease, ischemic stroke history, systemic embolization, major bleeding, intracranial hemorrhage (ICH), and CHA_2_DS_2_‐VASc (0–1 and ≥2) and HAS‐BLED (0–2 and ≥3) risk scores. Subgroup analyses were calculated. In addition, IPTW adjustment was used to compare the risks of the major outcomes. A *p* value <0.05 was considered statistically significant. Statistical analyses were performed using SAS version 9.4 (SAS Institute).

## 3 RESULTS

**FIGURE 1:**
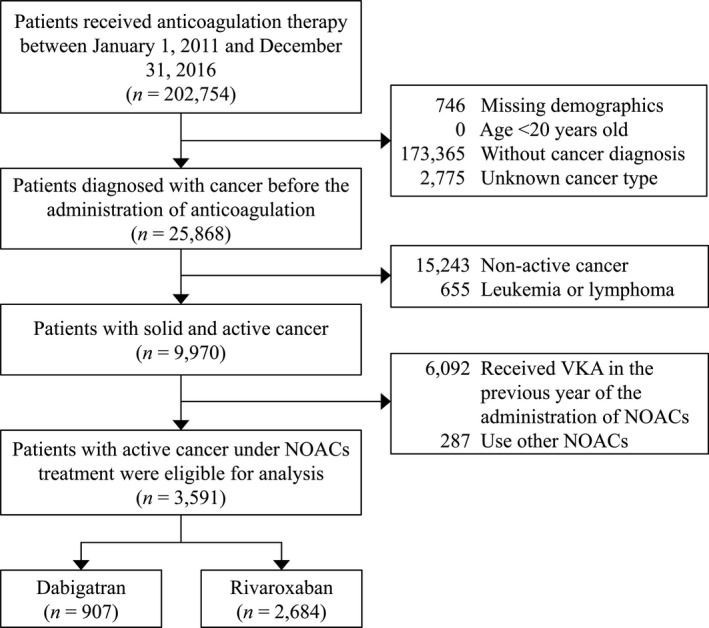
Flow chart for the inclusion of cancer patients on NOACs. NOAC, non‐vitamin K antagonist oral anticoagulants

There were 202,754 patients who received anticoagulation therapy between 2011 and 2016. Patients who took apixaban and edoxaban were excluded due to the short follow‐up period (apixaban, median: 8.8 ± 6.6 months) and small numbers (edoxaban, *N* = 287), which were insufficient for matching and outcome analysis. There were 3591 patients with active cancer eligible for analysis. Of these, 907 patients took dabigatran (approved in Taiwan on June 1, 2012) and 2684 patients took rivaroxaban (approved on February 1, 2013) (Figure 1). Before IPTW, most dabigatran prescriptions were for patients with coexisting AF or atrial flutter (81%), whereas those for rivaroxaban were for patients with coexisting VTE (61.3%). Compared with patients prescribed rivaroxaban, patients prescribed dabigatran were older (76.0 ± 9.0 vs. 69.7 ± 12.8 y) and had a higher prevalence of comorbidities, such as hypertension (80.9% vs. 62%), ischemic heart disease (32.6% vs. 22.2%), and heart failure (22.1% vs. 12.1%) (Table 1).

Regarding cancer types, dabigatran was prescribed mainly to patients with colorectal (24.3% vs. 17.8%) and male genital cancer (20.2% vs. 10.1%). In contrast, most rivaroxaban was prescribed to patients with lung cancer (13.1% vs. 19.3%) and female genital cancer (2.2% vs. 7.6%). Compared with rivaroxaban, angiotensin‐converting enzyme inhibitor (ACEi) and angiotensin receptor blockers (ARB) (58.5% vs. 43.3%), calcium channel blockers (38.5% vs. 31.9%), beta‐blockers (58.7% vs. 38.7%), and digoxin (22.8 vs. 9.9%) were more commonly prescribed to the dabigatran group. After matching with IPTW, the covariates were similar between the groups with absolute STD values <0.2 (Table 1).

### 3.1 Cancer‐related death and all‐cause mortality

**FIGURE 2:**
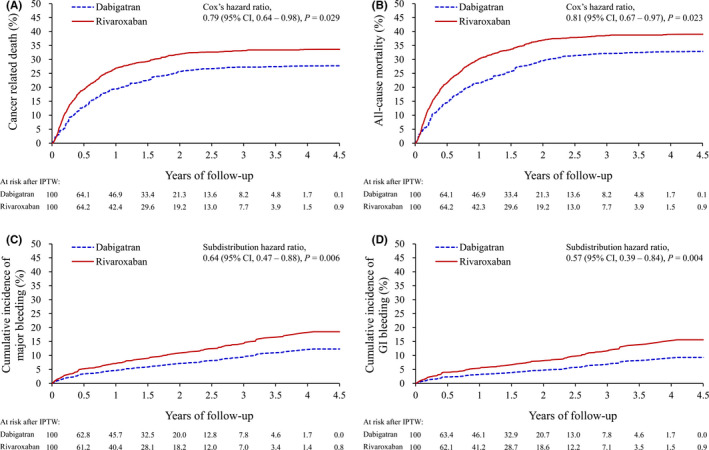
Cumulative event rates of cancer‐related death (A) and all‐cause mortality (B), and cumulative incidence function using the Fine and Gray method of major bleeding (C) and gastrointestinal bleeding (D) of patients with dabigatran or rivaroxaban treatments in the IPTW‐adjusted cohort. IPTW, inverse probability of treatment weighting

**FIGURE 3:**
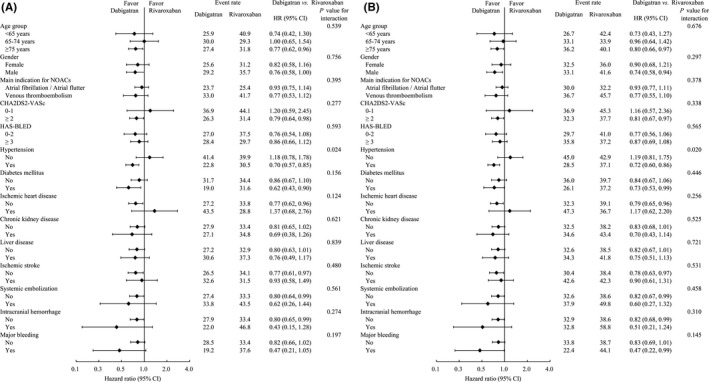
Pre‐specified subgroup analysis of cancer‐related death (A), all‐cause mortality (B)

During the entire observation period, there was a significantly lower risk of cancer‐related death in patients who received dabigatran than those who took rivaroxaban (27.7% vs. 33.6%; hazard ratio [HR] = 0.79, 95% confidence interval [CI] = 0.64–0.98; *p* = 0.029) (Table 2, Figure 2A). This observed effect on cancer‐related death was consistent across cancer stages (*P* for interaction =0.305; Table S3). The specific types of cancer with dabigatran‐associated lower cancer‐related death were colorectal (HR = 0.61, 95% CI = 0.41–0.90; *p* = 0.014), breast (HR = 0.43, 95% CI = 0.20–0.93; *p* = 0.033), male genital organ (HR = 0.54, 95% CI = 0.32–0.91; *p* = 0.020), and urinary tract cancers (HR = 0.45, 95% CI = 0.22–0.94; *p* = 0.034) (Table S4). Subgroup analysis showed that the observed effect was consistent in all variables, except hypertension (*P* for interaction =0.024; Figure 3A).

**TABLE 2:** Follow‐up outcomes of patients under dabigatran and rivaroxaban treatment after IPTW‐adjusted

Follow up length/Outcome	Event rate		Dabigatran vs. Rivaroxaban	
	Dabigatran	Rivaroxaban	HR (95% CI)^a^	*p* value
3 months follow‐up				
Cancer related death	7.7%	12.2%	0.61 (0.36–1.02)	0.059
Secondary outcomes				
All‐cause mortality	8.9%	13.7%	0.63 (0.40–0.99)	0.048
Major bleeding	1.1%	3.9%	0.28 (0.14–0.57)	<0.001
Gastrointestinal bleeding	0.7%	2.9%	0.25 (0.11–0.56)	<0.001
6 months follow‐up				
Cancer related death	12.8%	19.2%	0.66 (0.45–0.96)	0.028
Secondary outcomes				
All‐cause mortality	14.5%	21.5%	0.66 (0.47–0.93)	0.018
Major bleeding	2.5%	5.5%	0.45 (0.25–0.81)	0.008
Gastrointestinal bleeding	1.7%	4.2%	0.40 (0.19–0.85)	0.017
9 months follow‐up				
Cancer related death	16.6%	23.4%	0.70 (0.52–0.95)	0.023
Secondary outcomes				
All‐cause mortality	18.5%	26.3%	0.70 (0.53–0.92)	0.011
Major bleeding	2.7%	6.1%	0.45 (0.26–0.77)	0.004
Gastrointestinal bleeding	1.7%	4.6%	0.38 (0.19–0.78)	0.009
1‐year follow‐up				
Cancer related death	19.4%	26.9%	0.71 (0.54–0.93)	0.012
Secondary outcomes				
All‐cause mortality	21.5%	30.2%	0.70 (0.54–0.90)	0.005
Major bleeding	3.5%	6.7%	0.53 (0.33–0.83)	0.006
Gastrointestinal bleeding	2.3%	5.1%	0.46 (0.26–0.83)	0.009
At the end of the follow‐up				
Cancer related death	27.7%	33.6%	0.79 (0.64–0.98)	0.029
Secondary outcomes				
All‐cause mortality	32.9%	39.1%	0.81 (0.67–0.97)	0.023
Major bleeding	6.2%	9.6%	0.64 (0.47–0.88)	0.006
Gastrointestinal bleeding	4.3%	7.5%	0.57 (0.39–0.84)	0.004

Abbreviations: CI, confidence interval; HR, hazard ratio; IPTW, inverse probability of treatment weighting.

^a^ Estimated using the subdistribution hazard model which considered all‐cause death as a competing risk.

Regarding all‐cause mortality, there was also a significantly lower risk in patients with dabigatran compared to that of patients with rivaroxaban (32.9% vs. 39.1%; HR = 0.81, 95% CI = 0.67–0.97; *p* = 0.023) (Table 2, Figure 2B). This observed effect on all‐cause mortality was consistent across cancer stages (*P* for interaction =0.425; Table S3). The specific types of cancer that had seemed to have lower cancer‐related death associated with dabigatran uses were colorectal (HR = 0.64, 95% CI = 0.45–0.92; *p* = 0.014), male genital (HR = 0.60, 95% CI = 0.40–0.92; *p* = 0.019), and urinary tract cancers (HR = 0.40, 95% CI = 0.20–0.81; *p* = 0.010) (Table S4). However, these statistical results are offered post hoc and as an exploratory analysis. Therefore, we present these results as supplemental information and not as confirmation. Moreover, the subgroup analysis showed that the lower risks of cancer‐related death and all‐cause mortality associated with dabigatran compared with rivaroxaban were consistent across different levels of the subgroup variables, except for hypertension (*P* for interaction =0.020; Figure 3B).

### 3.2 Bleeding events between dabigatran and rivaroxaban groups

During the entire observation period, there was a significantly lower risk of major bleeding in patients taking dabigatran compared with rivaroxaban (6.2% vs. 9.6%; subdistribution hazard ratio [SHR] = 0.64, 95% CI = 0.47–0.88; *p* = 0.006) (Table 2, Figure 2C). This observed effect on major bleeding was consistent across cancer stages (*P* for interaction =0.088; Figure 3). The subgroup analysis showed that the observed effect was consistent in all variables, except previous ICH (*P* for interaction =0.021; Figure S1A).

Regarding GI bleeding, there was also a significantly lower risk in patients with dabigatran compared with that of patients with rivaroxaban (4.3% vs. 7.5%; SHR = 0.57, 95% CI = 0.39–0.84; *p* = 0.004) (Table 2, Figure 2D). This observed effect on GI bleeding was significantly more obvious in later stages of cancer (*P* for interaction =0.014; Figure 3). The subgroup analysis showed that the observed effect was consistent in all variables, except for age and HAS‐BLED score (*P* for interaction =0.018 and 0.045, respectively; Figure S1B).

## 4 DISCUSSION

To the best of our understanding, the current investigation on the impact of different classes of NOACs (dabigatran and rivaroxaban) on cancer survival is the first. Preclinical research has shown that coagulation and thrombosis play essential roles in cancer progression and spread at levels of thrombin and factor Xa regulation.^25^, ^26^ Therefore, thrombin and factor Xa inhibition seem to be critical steps in modulating cancer metastasis and progression.^25^, ^26^ The improvement of cancer survival has been reported by anticoagulation with LMWH use.^27^ However, NOACs, such as warfarin or apixaban, do not exhibit survival benefits in cancer patients at the cost of bleeding risk.^28^ It is hypothesized that the observed effects could result from the differential inhibition of the coagulation pathway, in which dabigatran acts directly on thrombin and avoids prothrombin feedback activation as rivaroxaban does. Besides, the major bleeding risk of rivaroxaban in this study was numerically higher (9.6% vs. 2.0%) than that with the prophylactic dosage (10 mg once daily for 180 days) reported by Khorana et al.,^14^ and the major bleeding risk of dabigatran (6.2%). In addition, rivaroxaban was comparable to other factor Xa inhibitors within the therapeutic dosage (1.1%–6.9%).^13^, ^15^

The antitumor benefits of anticoagulants have been debated for several decades. Previous studies revealed positive effects on cancer survival in randomized controlled trials. Also, the survival benefits of anti‐coagulations in cancer patients without venous thrombosis may be partly explained by the heterogeneity of designs of studies, types, and stages of cancers, therapeutic regimens, and classes, doses, and duration of the anticoagulants.^29^, ^30^ Our study adopted IPTW to minimize the selection bias between the dabigatran and rivaroxaban arm and showed clinically meaningful survival benefits compared with previous studies.^29^, ^30^ Patients with colorectal cancer, male genital organ cancers, and urinary tract cancer seemed to have a lower risk of cancer‐related death associated with dabigatran use (Table S4), which could be related to the strong expression of thrombin level in these malignancies.^31^ In addition, patients with female and male genital organs who received dabigatran showed differential effects on cancer survival, which could be partly explained by the gender difference in the blood coagulation system.^32^

## 5 LIMITATIONS

There are several limitations when using epidemiologic data from the national insurance database for studies. First, using ICD‐9‐CM and ICD‐10 codes for patient screening may miss certain cases for conditions not coded correctly. Second, the heterogeneity of the selection criteria and management between active cancers receiving standard treatment plus dabigatran and standard treatment plus rivaroxaban may lead to difficulties interpreting the results, especially the imbalance of indication of NOACs, as AF/flutter, venous thromboembolism, and underlying comorbidities. Moreover, a strength of this study is that actions were taken to reduce these potential biases. Indeed, the data on cancer diagnosis, initial cancer stage, corresponding treatment, cancer‐related death, and all‐cause mortality were extracted from cross‐links to a national level data of cancer registry and death with insurance covering 99.7% of the whole population. Also, a propensity score based on the IPTW adjustment was used to reduce the confounding bias and imbalances in covariates, potentially estimating treatment effects similar to randomized trials. Third, regarding bleeding events, minor bleeding events (i.e., gum bleeding) may not require medical attention and would result in undercoded (underreported) information as adverse events. Therefore, in our study, only major bleeding events requiring blood transfusion >2 Units, life‐threatening bleeding or vital organ hemorrhage, such as ICH and GI bleeding, which necessitated intervention, treatment in the emergency room, or during hospitalization, were studied as outcomes. Finally, this study was conducted in a primarily ethnic homogenous population, and whether these findings apply to other populations warrants further studies.

## 6 CONCLUSION

In cancer patients with VTE or AF, the use of dabigatran may be associated with a lower risk of cancer‐related death and all‐cause mortality compared with rivaroxaban. Further studies are warranted to confirm these findings.

## ETHICS STATEMENT

All procedures followed were in accordance with the ethical standards of the responsible committee on human experimentation by Chang Gung Memorial Hospital, Taiwan, and with the Helsinki Declaration of 1975, as revised in 2000. The de‐identified medical and health information can provide analyzable data through which research can be conducted and informed consent is waived. The current study is approved by the Institutional Review Board (IRB) at Chang Gung Memorial Hospital, Chiayi Branch (IRB No. 201901482B1).

## CONFLICT OF INTEREST

None.

## Supporting information

Supplementary Material

## Data Availability

Derived data supporting the findings of this study are available from the corresponding author upon request.
