# Supplementary material for: Mortality associated with the use of non‐vitamin K antagonist oral anticoagulants in cancer patients: Dabigatran versus rivaroxaban
Source: Cancer Med. 2021 Aug 31;10(20):7079–88. doi: 10.1002/cam4.4241 (PMC8525135; doi:10.1002/cam4.4241)
Supplement: Supplementary file 1 — Supplementary Material [file CAM4-10-7079-s001.docx]

**Supplemental Materials**

**Supplemental Figure 1.** Pre-specified subgroup analysis of major bleeding (A) and gastrointestinal bleeding (B).

**Supplemental Table 1.** ATC code of drugs use in this study

**Supplemental Table 2.** Disease code use in this study

**Supplemental Table 3.** The trend test between Dabigatran and Rivaroxaban on outcomes across different initial cancer stages after IPTW-adjusted

**Supplemental Table 4.** The effect between Dabigatran and Rivaroxaban on all-cause mortality and cancer-related death stratified by cancer types after IPTW-adjusted

**Supplemental Figure 1.** Pre-specified subgroup analysis of major bleeding (A) and gastrointestinal bleeding (B).
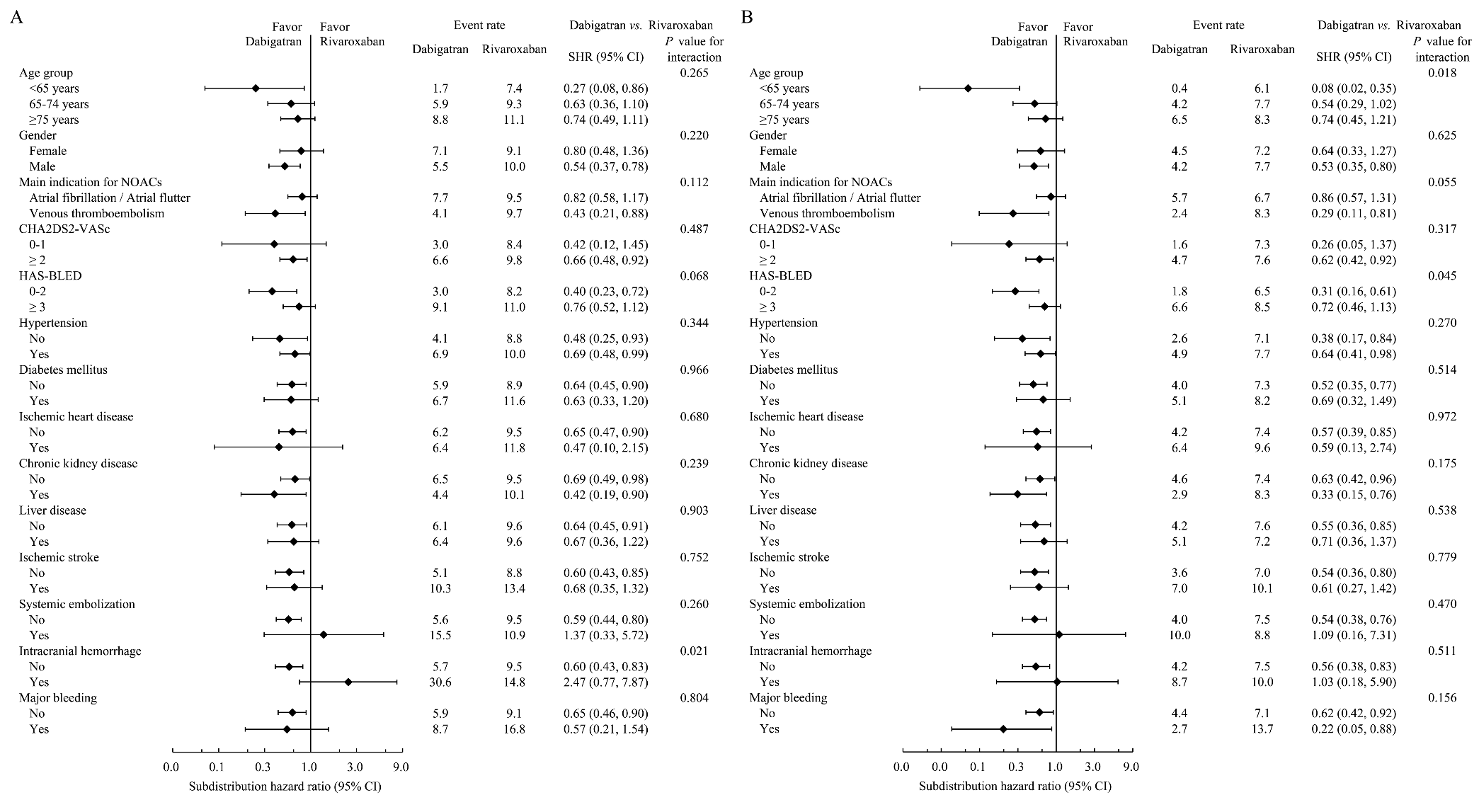


**Supplemental Table 1.** ATC code of drugs use in this study

| **Drug** | **ATC code** |
| --- | --- |
| ACEI/ARB | C09 |
| Non-dihydropyridine CCB | C08DA01, C08DB01 |
| Dihydropyridine CCB | C08CA01, C08CA02, C08CA03, C08CA04, C08CA05, C08CA06, C08CA07, C08CA08, C08CA09, C08CA12, C08CA13, C08CA15 |
| β-blocker | C07 |
| Diuretics | C03 |
| Spironolactone | C03DA01 |
| Digoxin | C01AA05 |
| Statin | C10AA |
| DPP4i | A10BH |
| Metformin | A10BA |
| Sulfonylurea | A10BB |
| Thiazolidinedione | A10BG02, A10BG03 |
| Insulin | A10A |
| NSAIDs or COX-2 | M01AA, M01AB, M01AC, M01AE, M01AG, M01AX, M01AH |
| Steriod | H02AB, H02AB01, H02AB02, H02AB04, H02AB05, H02AB06, H02AB08, H02AB09, H02AB10 |
| Antiplatelets | B01AC |
| PPI IV form | A02BC01, A02BC02, A02BC03, A02BC05 |

ATC, Anatomical Therapeutic Chemical; ACEI, angiotensin converting enzyme inhibitor; ARB, angiotensin II receptor blockers; CCB, calcium channel blocker; DPP4i, dipeptidyl peptidase 4 inhibitors; NSAID, non-steroidal anti-inflammatory drug; COX-2, Cyclooxygenase-2; PPI, proton pump inhibitor; IV, intravenous.

**Supplemental Table 2.** Disease code use in this study

| **Select study cohort** | **ICD-O-3** | **ICD-9-CM** | **ICD-10-CM** |
| --- | --- | --- | --- |
| Cancer | C00-D49 |  |  |
| Hematologic cancer | M-98003~M-99643, M-99803~M-99893, M-95903~M-97293, M-97503~M-97583, M-97643 |  |  |
| **Indication for NOACs** |  |  |  |
| Atrial fibrillation / Atrial flutter |  | 427.3x | I48 |
| Venous thromboembolism |  | 415.xx, 452.xx, 453.xx, 673.8x, 415.1x | I81, I82, I26.9, I26.09, I27.82, T800XXA, T81718A, T8172XA, T82817A, T82818A |
| **Cancer types** |  |  |  |
| Colorectal | C18-C20 |  |  |
| Lung | C33-C34 |  |  |
| Breast | C50 |  |  |
| Male genital organs | C60-C63 |  |  |
| Female genital organs | C51-C58 |  |  |
| Liver | C22 |  |  |
| Urinary | C64-C68 |  |  |
| Head and neck cancers | C05-C06, C09-C10, C12-C13 |  |  |
| Non-colorectal gastrointestinal cancers | C15-C17, C21, C23-C26, C48 |  |  |
| **Comorbidities** |  |  |  |
| Hypertension |  | 401.xx-405.xx with any anti-hypertension drugs | I10-I15, N262 with any anti-hypertension drugs |
| Diabetes mellitus |  | 250.xx with any oral hypoglycemic drugs and insulin | E08-E13 with any oral hypoglycemic drugs and insulin |
| Dyslipidemia |  | 272.0-272.4 with any anti-hyperlipidemia drugs | E77, E780, E781, E782, E783, E784, E785, E786, E881, E753, E755, E882, E756, E789, E7521, E7522, E7524, E7130, E7879, E7881, E7889, E8889, E7870 with any anti-hyperlipidemia drugs |
| Ischemic heart disease |  | 410.xx-414.xx | I20-I24 |
| Heart failure |  | 428.xx | I50 |
| Old myocardial infarction |  | 410.xx, 412.xx | I21-I22 |
| Gout |  | 274.xx | M10, M1A.0, M1A.2, M1A.3, M1A.4, M1A.9, N20.0 |
| Chronic obstructive pulmonary disease |  | 491.xx, 492.xx, 496.xx | J41-J44 |
| Peripheral artery disease |  | 440.xx, 441.xx, 443.xx, 444.0x, 444.8x, 447.8x, 447.9x, 093.0, 437.3, 444.22, 447.1, 557.1, 557.9, V434 | I70, I71, I73, I75, I771, I790, I791, I792, I773, I779, I798, K551, K558, K559, Z958, Z959, I743, I744, I745, I748, I740, I7789 |
| Chronic kidney disease |  | 580.xx-589.xx, 403.xx-404.xx, 016.0x, 095.4x, 236.9x, 250.4x, 274.1x, 442.1x, 447.3x, 440.1x, 572.4x, 642.1x, 646.2x, 753.1x, 283.11, 403.01, 404.02, 446.21 | A1811, D593, E102, E112, E132, I12, I13, K767, M103, M310, N00, N01, N02, N03, N04, N05, N06, N07, N08, N14, N150, N158, N159, N16, N171, N172, N18, N19, N200, N25, N261, N269, N27, Q61 |
| Alcohol-use disorder |  | 571.2 | K70, F1010, F1012, F1014, F1015, F1018, F1019, F1020, F1021, F1022, F1023, F1024, F1025, F1026, F1027, F1028, F1029, F1092, F1094, F1095, F1096, F1097, F1098, F1099, G621, I426, T510X1A, T510X2A, T510X3A, T510X4A, Z658 |
| Liver disease |  | 070.xx, 456.0-456.2, 570.xx, 571.xx, 572.2-572.8, 573.xx, V42.7 | B15, B16, B17, B18, B19, I85, K70, K71, K72, K73, K74, K752, K753, K754, K758, K759, K76, K77, Z944 |
| **Event history and outcomes** |  |  |  |
| Ischemic stroke |  | 433.xx-437.xx | I66, I65.1, I65.0, I65.8, I65.9, I63.6, I63.8, I63.9, G45.0, G45.8, G45.1, G45.2, G46.0, G46.1, G46.2, G45.9, G45.4, G46.3, G46.4, G46.5, G46.6, G46.7, G46.8, I67.0, I67.1, I67.2, I67.4, I67.5, I67.6, I67.7, I67.9, I68.0, I68.2, I68.8 |
| Systemic embolization |  | 362.3x, 444.9x, 444.22, 444.81, 444.21, 362.30, 362.34, 593.81, 444.89, 557.0, 557.9, 557.1 | I74, I75, I23.6, G45.3, H34.0, H34.9, K55.0, K55.1, K55.8, K55.9, N28.0 |
| Intracranial hemorrhage |  | 430.xx-432.xx | I60-I62 |
| Major bleeding (including gastrointestinal bleeding) |  | 430, 431, 432.0, 432.1, 432.9, 852.0, 852.2, 852.4, 853.0, 530.7, 531, 531.2, 531.4, 531.6, 532, 532.2, 532.4, 532.6, 533, 533.2, 533.4, 533.6, 534, 534.2, 534.4, 534.6, 569.3, 535.01, 535.11, 535.21, 535.31, 535.41, 535.51, 535.61, 535.71, 537.83, 537.84, 562.02, 562.03, 562.12, 562.13, 569.85, 578, 336.1, 363.6, 372.72, 376.32, 377.42, 379.23, 593.81, 866.01, 866.02, 866.11, 866.12, 719.1, 729.92, 423.0, 772.5 | I60, I61, I62, K25, K26, K27, K28, G95.1, H11.3, H31.3, H43.1, I31.2, K22.6, K62.5, K92.0, K92.1, K92.2, M25.0, N28.0, P54.4, H05.23, H47.02, K29.01, K29.21, K29.31, K29.31, K29.41, K29.51, K29.61, K29.71, K29.81, K29.91, K31.82, K52.81, K55.21, K56.60, K57.01, K57.11, K57.13, K57.21, K57.31, K57.33, K57.81, K57.91, K57.93, M79.81, K31.811, S06.340A, S06.341A, S06.342A, S06.343A, S06.344A, S06.345A, S06.346A, S06.347A, S06.348A, S06.349A, S06.350A, S06.351A, S06.352A, S06.353A, S06.354A, S06.355A, S06.356A, S06.357A, S06.358A, S06.359A, S06.360A, S06.361A, S06.362A, S06.363A, S06.364A, S06.365A, S06.366A, S06.367A, S06.368A, S06.369A, S06.4X0A, S06.4X1A, S06.4X2A, S06.4X3A, S06.4X4A, S06.4X5A, S06.4X6A, S06.4X7A, S06.4X8A, S06.4X9A, S06.5X0A, S06.5X1A, S06.5X2A, S06.5X3A, S06.5X4A, S06.5X5A, S06.5X6A, S06.5X7A, S06.5X8A, S06.5X9A, S06.6X0A, S06.6X1A, S06.6X2A, S06.6X3A, S06.6X4A, S06.6X5A, S06.6X6A, S06.6X7A, S06.6X8A, S06.6X9A, S31.001A, S31.001A, S37.011A, S37.012A, S37.019A, S37.019A, S37.021A, S37.022A, S37.029A, S37.029A, S37.031A, S37.032A, S37.039A, S37.039A, S37.041A, S37.042A, S37.049A, S37.049A, S37.051A, S37.052A, S37.059A, S37.059A |

ICD-9-CM, International Classification of Diseases, Ninth Revision, Clinical Modification; ICD-O-3, International Classification of Diseases for Oncology, Third Edition; ICD-10-CM, International Classification of Diseases, Tenth Revision, Clinical Modification.

**Supplemental Table 3.** The trend test between Dabigatran and Rivaroxaban on outcomes across different initial cancer stages after IPTW-adjusted

|  | Dabigatran |  | Rivaroxaban |  | Dabigatran *vs.* Rivaroxaban | | |
| --- | --- | --- | --- | --- | --- | --- | --- |
| IPTW-adjusted analysis | Event rate |  | Event rate |  | HR (95% CI) | *P* value |  |
| Cancer related death |  |  |  |  |  |  |  |
| Stage 0-2 | 20.8% |  | 21.8% |  | 0.93 (0.50, 1.72) | 0.819 |  |
| Stage 3 | 28.7% |  | 36.5% |  | 0.66 (0.45, 0.95) | 0.027 |  |
| Stage 4 | 74.4% |  | 57.0% |  | 1.23 (0.86, 1.75) | 0.264 |  |
| *P* of trend test |  |  |  |  | - | 0.305 |  |
| All-cause mortality |  |  |  |  |  |  |  |
| Stage 0-2 | 30.8% |  | 29.5% |  | 1.02 (0.65, 1.61) | 0.934 |  |
| Stage 3 | 31.1% |  | 41.8% |  | 0.62 (0.44, 0.88) | 0.008 |  |
| Stage 4 | 79.0% |  | 61.4% |  | 1.21 (0.86, 1.70) | 0.284 |  |
| *P* of trend test |  |  |  |  | - | 0.425 |  |
| Major bleeding# |  |  |  |  |  |  |  |
| Stage 0-2 | 8.1% |  | 9.5% |  | 0.82 (0.47, 1.42) | 0.472 |  |
| Stage 3 | 6.6% |  | 8.6% |  | 0.71 (0.32, 1.55) | 0.386 |  |
| Stage 4 | 2.9% |  | 8.2% |  | 0.30 (0.12, 0.77) | 0.012 |  |
| *P* of trend test |  |  |  |  | - | 0.088 |  |
| GI bleeding# |  |  |  |  |  |  |  |
| Stage 0-2 | 5.9% |  | 6.1% |  | 0.93 (0.48, 1.82) | 0.835 |  |
| Stage 3 | 3.5% |  | 7.7% |  | 0.42 (0.18, 0.95) | 0.037 |  |
| Stage 4 | 2.0% |  | 7.2% |  | 0.24 (0.09, 0.60) | 0.002 |  |
| *P* of trend test |  |  |  |  | - | 0.014 |  |

IPTW, inverse probability of treatment weighting; HR, hazard ratio; CI, confidence interval;

# Estimated using the subdistribution hazard model which considered all-cause death as a competing risk.

**Supplemental Table 4.** The effect between Dabigatran and Rivaroxaban on all-cause mortality and cancer-related death stratified by cancer types after IPTW-adjusted

|  | Dabigatran |  | Rivaroxaban |  | Dabigatran *vs.* Rivaroxaban | |
| --- | --- | --- | --- | --- | --- | --- |
| IPTW adjusted analysis | Event rate |  | Event rate |  | HR (95% CI) | *P* value |
| **Cancer related death** |  |  |  |  |  |  |
| Colon rectal | 21.0% |  | 31.6% |  | 0.61 (0.41, 0.90) | 0.014 |
| Lung | 49.1% |  | 59.1% |  | 0.92 (0.64, 1.33) | 0.662 |
| Breast | 4.6% |  | 11.5% |  | 0.43 (0.20, 0.93) | 0.033 |
| Male genital organs | 11.9% |  | 20.0% |  | 0.54 (0.32, 0.91) | 0.020 |
| Female genital organs | 62.2% |  | 45.9% |  | 1.40 (0.62, 3.14) | 0.421 |
| Liver | 54.0% |  | 40.8% |  | 1.19 (0.74, 1.90) | 0.484 |
| Urinary tract | 15.9% |  | 29.3% |  | 0.45 (0.22, 0.94) | 0.034 |
| Head and neck (including oral cancer) | 14.4% |  | 28.8% |  | 0.63 (0.26, 1.53) | 0.310 |
| Digestive organs | 48.8% |  | 62.6% |  | 0.61 (0.28, 1.33) | 0.211 |
| Others | 23.0% |  | 23.9% |  | 0.85 (0.48, 1.50) | 0.567 |
| **All-cause mortality** |  |  |  |  |  |  |
| Colon rectal | 26.0% |  | 36.7% |  | 0.64 (0.45, 0.92) | 0.014 |
| Lung | 51.6% |  | 60.7% |  | 0.94 (0.66, 1.35) | 0.737 |
| Breast | 14.3% |  | 17.8% |  | 0.87 (0.53, 1.42) | 0.570 |
| Male genital organs | 20.4% |  | 30.6% |  | 0.60 (0.40, 0.92) | 0.019 |
| Female genital organs | 63.8% |  | 49.5% |  | 1.32 (0.60, 2.91) | 0.496 |
| Liver | 59.4% |  | 43.2% |  | 1.21 (0.77, 1.91) | 0.403 |
| Urinary tract | 16.8% |  | 35.2% |  | 0.40 (0.20, 0.81) | 0.010 |
| Head and neck (including oral cancer) | 16.4% |  | 36.4% |  | 0.57 (0.25, 1.28) | 0.171 |
| Digestive organs | 61.1% |  | 66.1% |  | 0.73 (0.34, 1.55) | 0.410 |
| Others | 24.3% |  | 31.3% |  | 0.68 (0.40, 1.17) | 0.162 |

IPTW, inverse probability of treatment weighting; HR, hazard ratio; CI, confidence interval.
